# A novel AI-enhanced microwave sensor employing defected ground structure for non-invasive glucose monitoring

**DOI:** 10.1038/s41598-026-40171-9

**Published:** 2026-02-19

**Authors:** Fikret Alpay Tekşen, Seda Aygül, Berker Çolak, Fatih Özkan Alkurt, Muharrem Karaaslan, Yakup Hameş, Edik Rafaliov, Tatjana Gric

**Affiliations:** 1 Department of Electronic Systems, VILNIUS TECH, Vilnius, Lithuania; 2 Department of Electrical and Electronics Engineering, Iskenderun Technical University, Hatay, Turkey; 3 Techschenlab Technology Inc. Iskenderun, Hatay, Turkey; 4 Aston Institute of Photonic Technologies, Aston University, Birmingham, B4 7ET UK; 5 Department of Electrical and Electronics Engineering, Sivas University of Science and Technology, Sivas, Turkey

**Keywords:** Non-invasive glucose monitoring, Microwave sensor, Defected ground structure, Microstrip antenna, Wearable sensor, Engineering, Health care

## Abstract

Diabetes is a chronic disease that affects millions of people worldwide and significantly reduces quality of life. One of the most critical aspects of managing this condition is the accurate, continuous, and reliable monitoring of blood glucose levels. Fluctuations in glucose concentration can lead to both short-term complications and long-term irreversible organ damage. Currently, traditional glucose monitoring methods rely mainly on blood samples obtained through finger-pricking. While these methods are accurate, their invasive nature reduces user comfort, causes pain, poses a risk of infection, and negatively affects patient adherence in the long run. In response to these limitations, non-invasive glucose monitoring technologies, particularly those based on microwave and radio frequency (RF) sensor systems, have gained increasing attention. However, most reported systems still face challenges in achieving high sensitivity and stability under realistic physiological conditions. In this study, we introduce a novel hexagonal microstrip patch antenna with a chaotic Defected Ground Structure (DGS) based on a Duffing chaotic attractor, specifically designed for non-invasive blood glucose sensing. Unlike conventional DGS-based sensors, our chaotic DGS approach enhances tissue penetration and significantly improves sensitivity to subtle dielectric variations caused by glucose concentration changes. The sensor, optimized for finger placement, operates in the 4–5 GHz range to ensure effective tissue coupling. Experimental validation using multi-layer tissue-mimicking phantoms demonstrated the sensor’s ability to differentiate clinically relevant glucose levels (50–200 mg/dL), achieving a high sensitivity of 0.950 MHz/(mg/dL).

## Introduction

Diabetes mellitus, a metabolic disorder characterized by chronic hyperglycemia resulting from defects in insulin secretion or action, has escalated into a global health crisis^1,2^. While Type 1 diabetes is defined by an absolute insulin deficiency from the autoimmune destruction of pancreatic β-cells^3^, Type 2 diabetes involves insulin resistance and progressive β-cell dysfunction^4^. Chronic hyperglycemia leads to severe microvascular complications, such as retinopathy and neuropathy, and macrovascular conditions, including cardiovascular disease^5^. Consequently, the regular monitoring of blood glucose levels (BGL) is a cornerstone of modern diabetes management guidelines, which emphasize patient-centered, evidence-based care to mitigate these risks and improve quality of life^6,7^.

The conventional method for BGL monitoring, self-monitoring via finger-pricking (SMBG), presents significant challenges to patient adherence. Barriers such as pain, inconvenience, and financial cost are widely reported by patients and are strongly correlated with reduced testing frequency^8,9^. This poor compliance can lead to inadequate glycemic control, thereby increasing the risk of long-term complications^10^. These limitations have catalyzed extensive research into non-invasive blood glucose monitoring (NIBGM) technologies, which promise to eliminate pain, reduce infection risk, and improve patient adherence^11^. The NIBGM field encompasses a wide array of techniques, including optical spectroscopy, transdermal, and biofluid-based approaches, each facing unique challenges related to accuracy, calibration, and signal stability^12,13^.

Among these technologies, microwave and electromagnetic sensing have emerged as a particularly promising frontier^14^. This approach is based on the fundamental principle that the complex permittivity of blood exhibits a functional dependence on its glucose concentration^15^. The dielectric properties of biological tissues are influenced by multiple factors, including tissue type, frequency, and temperature, offering a rich data source for high-precision sensing applications^16,17^. Importantly, the microwave signals used in such biomedical devices are non-ionizing and operate well below the thermal effect limits established by international safety standards, ensuring their viability for continuous and safe monitoring^18,19^.

In recent years, significant engineering efforts have focused on enhancing the sensitivity, selectivity, and compactness of microwave sensors for NIBGM. State-of-the-art designs frequently employ metamaterial-based resonators, particularly Complementary Split-Ring Resonators (CSRRs), to confine the electromagnetic field and enhance sensitivity^20,21^. Another powerful technique is the use of a DGS, where geometric patterns are etched into the ground plane of antennas and sensors to achieve miniaturization, improved impedance matching, and a higher quality factor (Q-factor)^22^. Furthermore, to enhance the accuracy of glucose prediction from the complex data generated by these advanced sensors, machine learning (ML) algorithms such as Gaussian Process Regression and neural networks are being successfully integrated^23,24^.

While conventional DGS geometries like dumbbells or spirals have shown success in improving sensor performance, they often offer limited Q-factor enhancement and can be sensitive to fabrication tolerances. The potential of non-Euclidean, complex geometries to overcome these limitations remains largely unexplored. To address this opportunity, this study proposes a novel NIBGM sensor based on a microstrip patch antenna designed to operate in the 3–4 GHz frequency range. The core innovation of this sensor lies in its ground plane, which incorporates a novel DGS patterned from a Duffing chaotic attractor. It is hypothesized that this unique geometry, unlike conventional DGS shapes, creates a more intense and focused electromagnetic field, leading to a superior response to dielectric variations. The sensor is optimized for finger placement, and its performance is evaluated using multi-layer, tissue-mimicking phantoms that include epidermis, dermis, fat, and blood layers. The proposed design demonstrates strong potential for integration into wearable and portable medical devices for continuous, non-invasive diabetes management. This paper is organized as follows: Section II details the sensor design and methodology, Section III presents the simulation and measurement results, Section IV discusses the findings, and Section V concludes the study.

## Sensor design and methodology

The operating frequency band of the antenna was determined by considering the interaction of biological tissues with electromagnetic waves. The human body, particularly layers such as skin, fat, and blood, significantly attenuates and absorbs microwaves before they can penetrate deeply. This causes the signal to weaken within the superficial layers when very high frequencies are used, thereby preventing sufficient information from being obtained from deeper tissues. On the other hand, at lower frequencies, the increase in wavelength reduces the ability to distinguish small variations in dielectric properties, making it more difficult to detect changes in glucose concentration. Therefore, the frequency selection was made by balancing the need for adequate penetration depth with the requirement to sense dielectric contrast with high precision. In this context, the Industrial, Scientific, and Medical (ISM) band was chosen as the optimal solution. The antenna design operating in the ISM band was carried out by taking into account the skin depth effect, thus enabling the sensor to establish effective electromagnetic coupling with biological tissues while maintaining sensitivity to subtle changes in glucose levels. The design process commences with a hexagonal microstrip patch antenna, engineered to operate in the 4–5 GHz frequency range. The key innovation lies in the modification of the ground plane, where a standard ground is replaced with a novel DGS derived from the mathematical model of a Duffing chaotic attractor. This unique DGS is hypothesized to enhance the sensor’s quality factor (Q-factor) and localize the electromagnetic field interaction with the biological tissue. The sensor’s geometry was meticulously optimized using the full-wave electromagnetic simulator, CST Microwave Studio. Subsequent to the simulation phase, a physical prototype was fabricated on a Rogers RO5870 high-frequency substrate. The methodology is completed by an experimental validation framework, which involves S-parameter measurements of the fabricated sensor and its testing with multi-layer tissue-mimicking phantoms designed to replicate the dielectric properties of a human finger under varying glucose concentrations.

### Sensor geometry and a novel DGS

The proposed non-invasive glucose sensor is based on a hexagonally shaped microstrip patch antenna, which is specifically chosen to ensure conformal contact and efficient electromagnetic coupling with the surface of a human finger. The entire structure is designed on a 1.52 mm thick Rogers RO5870 substrate, which has a dielectric constant (ϵ_*r*_) of 2.33 and a loss tangent of 0.0012. The standard copper cladding thickness is 0.035 mm. The key innovation of this design is the strategic modification of the antenna’s ground plane to enhance its sensing capabilities. Instead of a conventional solid ground plane, half of the ground is etched with a novel DGS patterned from the phase-space portrait of a Duffing chaotic attractor.

The Duffing attractor^25^ is a non-linear dynamical system known for its chaotic behavior under certain parameter sets. Its dynamics are described by the second-order differential equation:

1 $$\frac{{{d^2}x}}{{d{t^2}}}+\delta \frac{{dx}}{{dt}}+\alpha x+\beta x=\gamma \cos (\omega t)$$

where *x* is the position, *t* is time, δ represents the damping coefficient, and the remaining terms describe the restoring force and the external driving force. For this work, the parameters were set to δ = 0.2, α = −1, β = 1, γ = 0.3, and ω = 1.2 to generate a chaotic trajectory. Equation 1 was solved numerically to generate a time-series solution for position *x*(*t*) and velocity $\dot {x}(t)$. The resulting $(x,\dot {x})$ pairs, when plotted in a 2D phase space, form the characteristic shape of the.

**Fig. 1:**
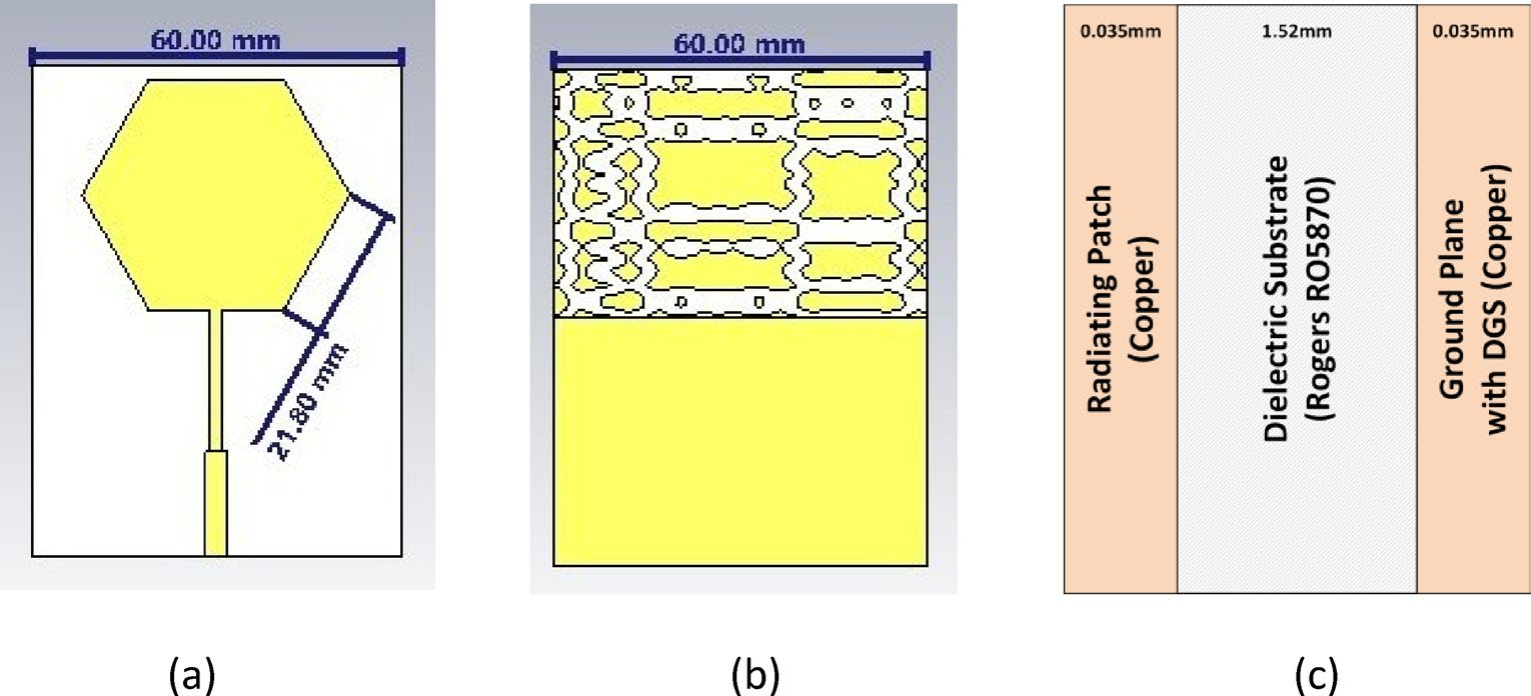
Geometry of the proposed chaotic DGS sensor: **a** top view showing the hexagonal patch (side length = 21.80 mm) and microstrip feedline. **b** Bottom view showing the chaotic DGS pattern on the partial ground plane. **c** Side view showing the substrate and conductor layers with their respective thicknesses (substrate height = 1.52 mm, copper thickness = 0.035 mm). The total width is 60.00 mm.

Duffing attractor. This continuous trajectory was then discretized and mapped into a conductive pattern to form the DGS, as illustrated in the sensor’s ground plane in Fig. 1b. This non- Euclidean, complex geometry is designed to create highly localized and intense electric fields, thereby increasing the sensor’s quality (Q) factor and its sensitivity to minute changes in the dielectric properties of the adjacent tissue.

The detailed geometry of the proposed sensor is depicted in Fig. 1. The overall width of the substrate is 60.00 mm, and the side length of the hexagonal radiating patch is 21.80 mm. These dimensions are optimized to achieve strong resonance in the target frequency band.

### Simulation setup

The electromagnetic performance of the proposed sensor is rigorously analyzed and optimized using the commercial full-wave simulation software, CST Studio Suite 2025. For the characterization of the sensor’s reflection coefficient (*S*_11_) over a wide frequency band, the Time Domain (Transient) solver is employed. The simulation is executed over a frequency range from 2 to 6 GHz.

To ensure the accurate modeling of radiation into free space, the boundary conditions for all six faces of the simulation domain (Xmin/max, Ymin/max, and Zmin/max) are defined as ‘Open (add space)’. The microstrip antenna is excited using a discrete waveguide port with a characteristic impedance of 50 Ω, positioned at the edge of the microstrip feedline.

A hexahedral mesh is utilized for the discretization of the computational domain. The mesh properties have been configured to balance simulation accuracy with computational resources, with the key settings being 4 cells per wavelength and 10 cells per maximum model box edge. The ’Fraction of maximum cell near to model’ is set to 20. These settings result in a total of 187,136 hexahedral mesh cells for the model.

**Fig. 2:**
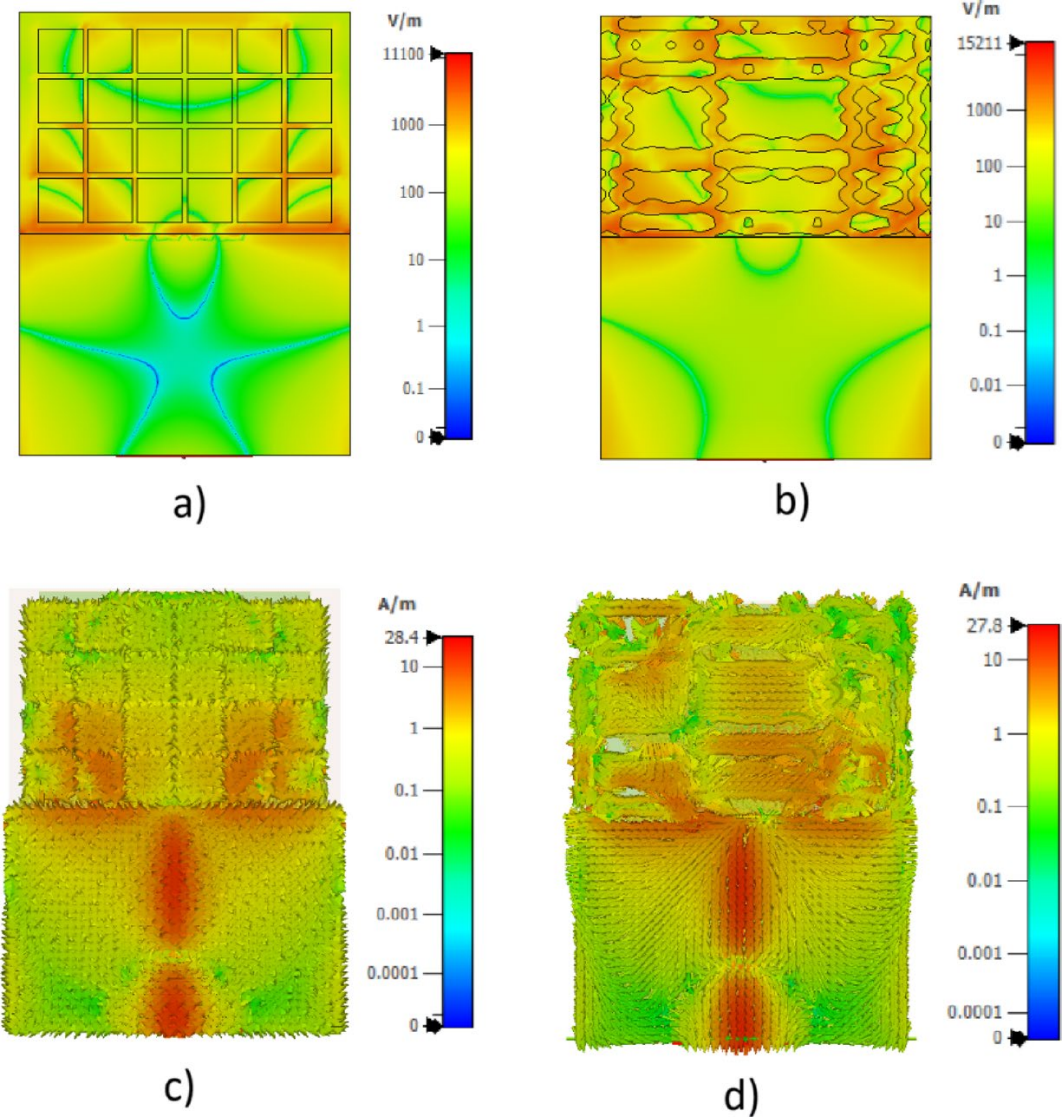
Comparison of near-field electric field magnitude and surface current density distributions for a conventional DGS and the proposed chaotic DGS with identical footprint and substrate parameters, evaluated at the resonance frequency. **a** Electric field distribution of the conventional DGS, **b** electric field distribution of the chaotic DGS, **c** surface current density of the conventional DGS, and **d** surface current density of the chaotic DGS.

Figure 2 provides a direct comparison of the near field electric field and surface current distributions between a conventional DGS and the proposed chaotic DGS with identical physical footprint. As observed in Fig. 2a, the conventional DGS exhibits a relatively smooth and spatially distributed electric field profile extending over a wider region of the ground plane. In contrast, the chaotic DGS in Fig. 2b produces stronger electric field localization concentrated around the chaotic slots and the sensing interface, indicating enhanced field confinement.

The surface current distributions further support this observation. As shown in Fig. 2c, the conventional DGS allows currents to spread more uniformly across the ground plane, resulting in shorter effective current paths. Conversely, the chaotic DGS in Fig. 2d enforces longer, tortuous current trajectories following the chaotic geometry, increasing the effective inductive loading and stored electromagnetic energy. This redistribution of current density suppresses undesired current leakage and radiation loss mechanisms.

The combined electric field confinement and extended current paths introduced by the chaotic DGS qualitatively explain the observed improvement in resonance sharpness and effective quality factor (Q), as confirmed by the S-parameter results presented in the previous section.

**Fig. 3:**
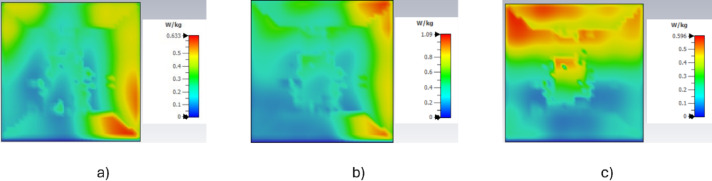
Specific absorption rate (SAR) distribution on the finger phantom at **a** 4 GHz, **b** 4.654 GHz and **c** 5 GHz.

In the Fig. 3, comparative analysis of the Specific Absorption Rate (SAR) distributions obtained on the finger phantom at different operating frequencies. In Fig. 3a, corresponding to 4 GHz, the maximum SAR value is approximately 0.63 W/kg, indicating relatively low energy absorption with localized regions of higher intensity. In Fig. 3b, at 4.654 GHz, which is the frequency where the antenna exhibits its best performance, the maximum SAR increases to about 1.09 W/kg. This increase is associated with improved electromagnetic coupling between the antenna and the phantom, resulting in a more effective and homogeneous power absorption while still remaining within safe limits. In Fig. 3c, at 5 GHz, the maximum SAR value decreases again to around 0.60 W/kg, suggesting reduced interaction and more superficial absorption compared to the optimum frequency. According to international safety standards, the maximum allowable localized SAR is 2 W/kg averaged over 10 g of biological tissue, as defined by the IEEE C95.1-2019 standard^26^. Since all obtained SAR values are well below this limit, the proposed antenna can be considered biologically safe. Moreover, the results confirm that at 4.654 GHz, the antenna achieves optimal performance while maintaining SAR levels within the accepted regulatory limits, demonstrating a favorable trade-off between efficiency and user safety.

### Fabrication and measurement setup

**Fig. 4:**
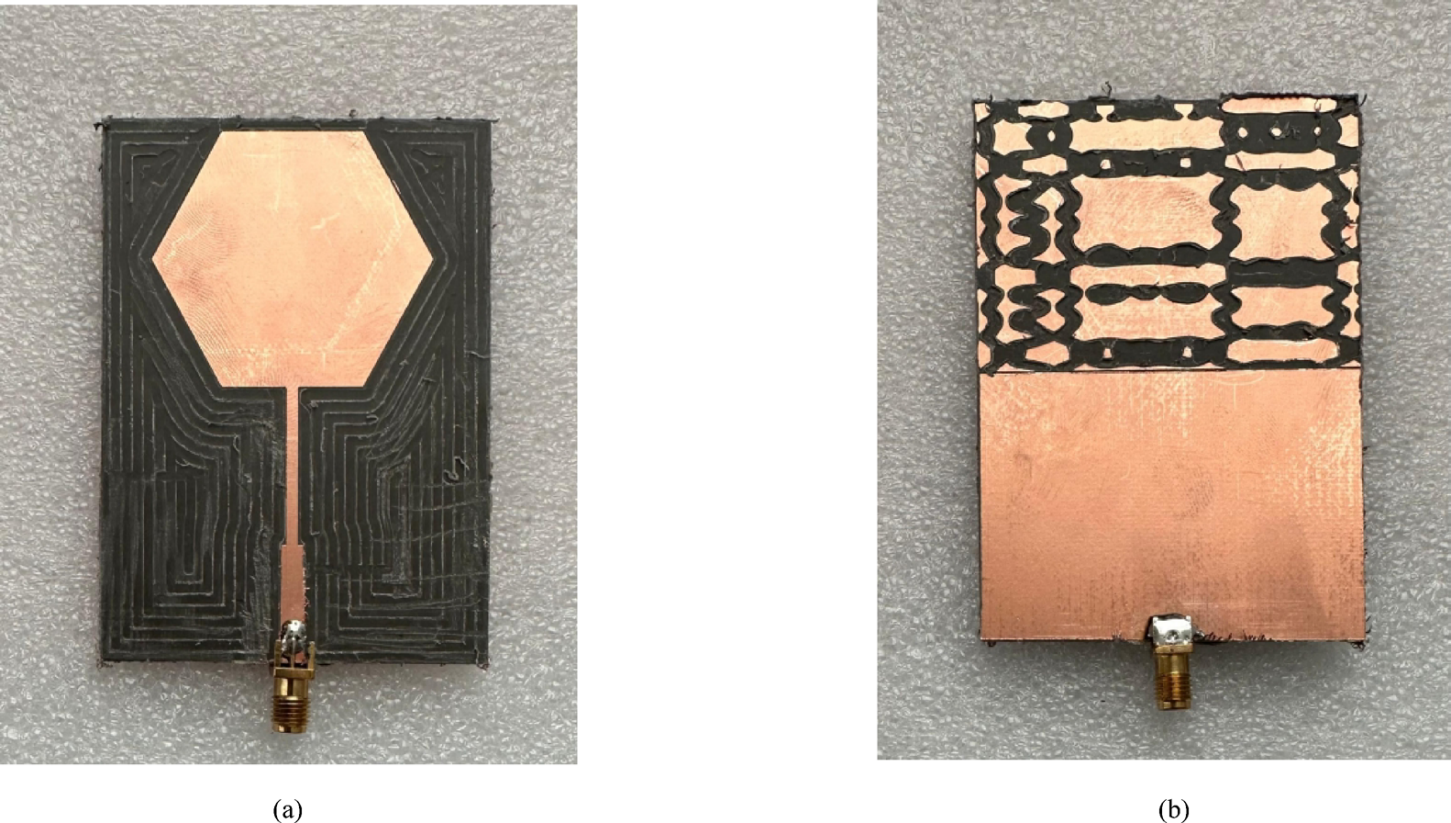
Photographs of the fabricated sensor prototype. **a** Top view of the prototype, **b** bottom view showing the DGS.

Following the successful simulation and optimization, a physical prototype of the proposed sensor is realized. The sensor is fabricated from a Rogers RO5870 laminate using an in-house PCB milling machine, which etched the hexagonal patch and the chaotic DGS pattern with high precision. Subsequently, a 50 Ω SMA (SubMiniature version A) connector is carefully mounted and affixed to the microstrip feedline via standard hand soldering to ensure a reliable interface for RF measurements. Photographs of the fabricated prototype are shown in Fig. 4.

**Fig. 5:**
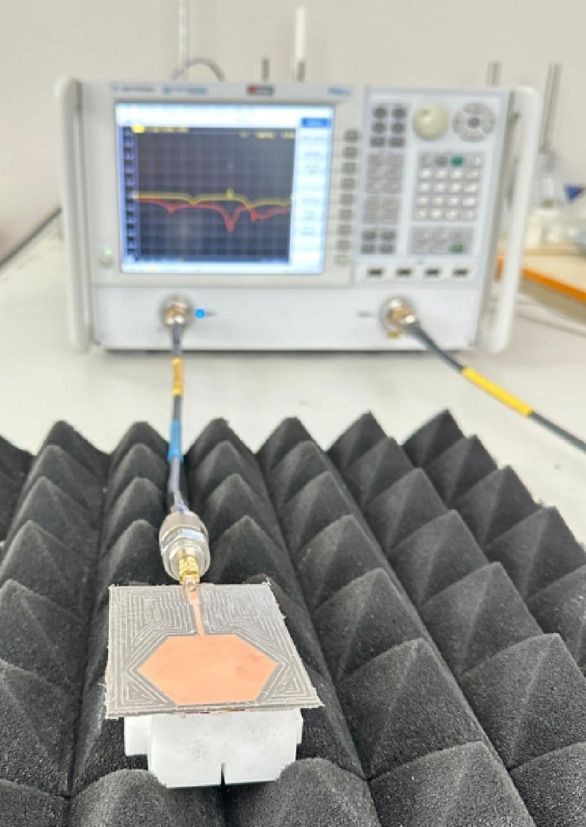
Photograph of the experimental measurement setup, showing the sensor under test placed on RF absorbing foams and connected to the Vector Network Analyzer (VNA).

The validation of the sensor’s performance is carried out by measuring its reflection coefficient (*S*_11_). The measurements are conducted in a temperature-controlled laboratory environment, maintained at 24 ± 1 °C. To minimize interference and unwanted reflections from the surroundings, the sensor is placed on a test fixture in front of RF absorbing foams. A Keysight PNA-L Vector Network Analyzer (VNA), model N5234A, is utilized for the S-parameter measurements, as shown in the experimental setup in Fig. 5. Prior to the measurements, a full one-port SOLT (Short-Open-Load-Thru) calibration is performed at the end of the RF cable to deembed the effects of the cable and establish an accurate reference plane at the sensor’s input port.

### Multi-layer phantom preparation

**Fig. 6:**
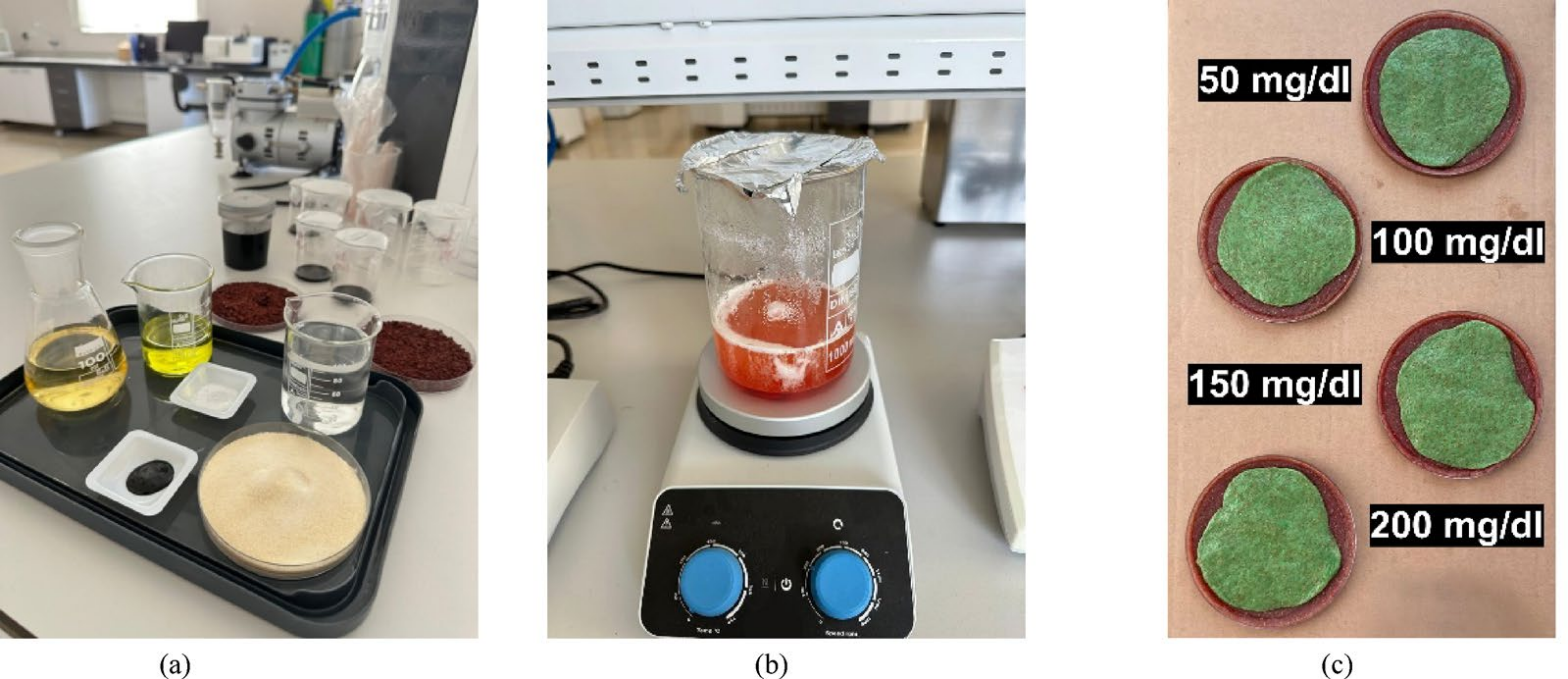
The phantom preparation process: **a** ingredients laid out for fabrication. **b** The mixture being heated and stirred. **c** The complete set of phantoms with varying glucose concentrations ready for measurement.

To experimentally validate the sensor’s sensitivity to glucose variations, a realistic multi- layer phantom mimicking the structure of a human finger is developed. The model consists of three primary layers: skin, fat, and blood. The recipes for the solid skin and fat tissue-mimicking phantoms are adapted from the well-established broadband phantom study by Yilmaz et al.^26^. The specific ingredients used for each phantom are detailed in Table 1. The complete fabrication process, from the raw ingredients to the final set of prepared phantoms, is visually documented in Fig. 6.

For the blood layer, aqueous solutions with varying glucose concentrations are prepared to simulate different glycemic states. Four distinct levels are tested: 50 mg/dL (hypoglycemia), 100 mg/dL (normoglycemia), 150 mg/dL, and 200 mg/dL (hyperglycemia). To ensure the accuracy of the experimental model, the dielectric properties (ε′) and (ε″) of all prepared phantoms are independently verified using a dielectric probe kit, and the results have been found to be in close agreement with the reference study^27^.

**Table 1:** Ingredients of the tissue-mimicking phantoms, adapted from^27^.

Ingredient (g)	Wet skin	Fat	Blood
Water	230.0	57.4	230.0
Gelatine	34.1	15.0	34.1
NaCl	1.4	0.0	1.2
Oil	75.0	329.6	15.0
Detergent	40.0	10.0	40.0
Food Coloring	1.3	0.0	0.0

The physical assembly of the phantom has been performed manually. The solid skin and fat phantoms are firstly prepared according to the recipes and then cut to the required dimensions using a utility knife to create a 1 mm thick skin layer and a 1.5 mm thick fat layer. For each measurement, the blood solution corresponding to a specific glucose level has been contained within a thin, electrically transparent plastic bag to form the 4 mm thick blood layer. After completing the design of the three-layer phantom model, the blood and glucose solution phantom is integrated into this structure. The primary rationale for this integration is to achieve a more realistic representation of biological conditions. In human tissue, one of the key determinants of the dielectric constant is the blood layer located beneath the fat tissue, whose properties vary depending on glucose concentration. Therefore, evaluating only the dielectric constant of the fat layer would be insufficient; it is essential to also account for the combined dielectric characteristics of blood and glucose solution. For this reason, the blood+glucose phantom has been merged with the fat layer to create a model that more closely mimics real biological conditions. Furthermore, this integration is carried out separately for each glucose level, and a total of four different blood glucose concentrations (50, 100, 150, and 200 mg/dL) have been evaluated. This mixed three-layer assembly is then carefully placed onto the sensor’s surface, with the skin layer in direct contact with the antenna, to perform the S-parameter measurements.

Furthermore, all phantoms used in this study were prepared at room temperature and all measurements were performed under constant environmental conditions. This approach was chosen to isolate the electromagnetic response associated with glucose concentration and to enable comparative evaluation of sensor performance. Nonetheless, the literature reports that, in microwave-band antenna-based wearable sensors temperature variations exert a limited but measurable effect on the resonance frequency^28^. Such thermal effects are generally slower and secondary compared with dielectric changes induced by glucose concentration. For translation to a wearable system, it is therefore important to account for temperature dependent frequency shifts: the antenna should be calibrated as a function of temperature, and the electromagnetic response to blood glucose model should be reformulated to include a temperature parameter. Practically, this can be achieved by measuring local skin temperature with an auxiliary sensor colocated with the antenna and using that temperature reading to correct or map the antenna response to the equivalent value at the reference temperature, thereby improving measurement accuracy under real world conditions.

## Results and analysis

This section presents the simulation and experimental results that characterize and validate the performance of the proposed non-invasive sensor. The analysis is presented in a structured manner, beginning with the fundamental validation of the design concept, followed by the verification of the fabricated prototype against the simulation model. Initially, the crucial impact of the chaotic DGS on the antenna’s performance is demonstrated by comparing the measured reflection coefficient of the proposed sensor with that of an identical antenna featuring a conventional solid ground plane. Subsequently, to validate the accuracy of the design process, the simulated *S*_11_ parameters of the optimized sensor are compared against the experimental measurements obtained from the fabricated prototype. Finally, the manuscript will present the sensor’s primary application: the experimental evaluation of its sensitivity to varying glucose concentrations using the multi-layer, tissue-mimicking phantoms described in the methodology.

### Impact of the chaotic DGS on antenna performance

The first step in the experimental validation is to demonstrate the effectiveness of the core design concept: the integration of the chaotic DGS. To achieve this, the measured performance of the proposed sensor is directly compared against a reference antenna. This reference antenna is identical in every aspect, including the hexagonal patch, feedline, and substrate, with the sole exception that it featured a conventional, solid ground plane instead of the patterned DGS.

**Fig. 7:**
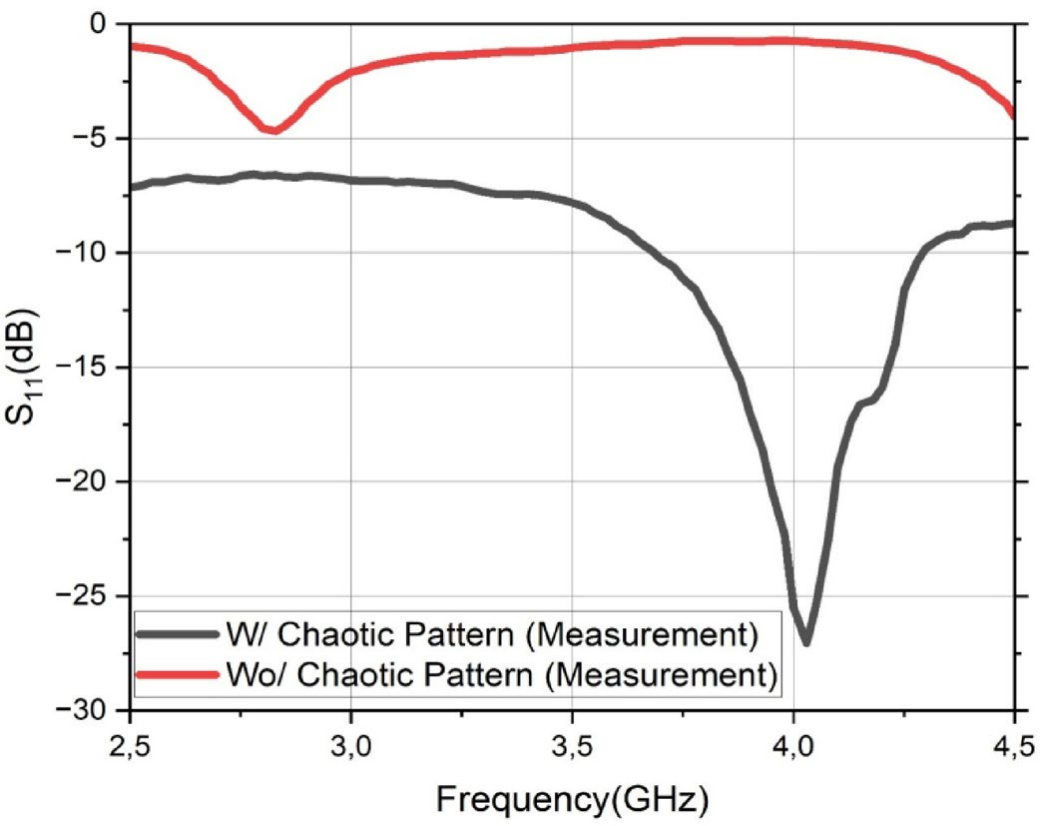
Measured reflection coefficient (*S*_11_) comparison between the proposed sensor with the chaotic DGS (black curve) and an identical reference antenna with a conventional solid ground plane (red curve).

The measured reflection coefficients (*S*_11_) for both antennas are presented in Fig. 7. The results provide a clear and compelling validation of the DGS’s function. The reference antenna (red curve) exhibits no resonant behavior across the entire measured frequency band from 2.5 to 4.5 GHz, with its *S*_11_ value remaining above − 5 dB. This indicates extremely poor impedance matching, rendering it unsuitable for any practical sensing or communication application.

In stark contrast, the introduction of the chaotic DGS (black curve) fundamentally transforms the antenna’s performance. A sharp and well-matched resonance is created at approximately 4.05 GHz, achieving a deep reflection coefficient of − 27.1 dB. This demonstrates that the chaotic DGS is not merely an incremental improvement but is the essential element responsible for creating the high-quality, sensitive resonance required for this application. This result confirms that the complex current paths induced by the DGS are critical for achieving the desired impedance matching and field confinement.

### Simulation and measurement agreement

To validate the accuracy of the electromagnetic model and the overall design methodology, the simulated reflection coefficient (*S*_11_) of the proposed sensor has been compared with the experimental measurements of the fabricated prototype. Figure 7 presents this comparison, overlaying the results for both the proposed sensor with the chaotic DGS and the reference antenna without it.

As depicted in the figure, there is a strong correlation between the simulated (green curve) and measured (black curve) results for the final sensor design. Both methodologies correctly predict that a strong resonance is formed near 4 GHz due to the DGS. However, minor discrepancies, which are common between simulation and real-world measurements, are observed. A slight frequency shift of approximately 100 MHz is present, with the simulated resonance occurring at 3.95 GHz and the measured resonance at 4.05 GHz. Furthermore, a notable difference is seen in the resonance depth; the measured prototype achieves a significantly better impedance matching of − 27.1 dB compared to the simulated prediction of − 18 dB.

These minor variations can be attributed to several factors. The frequency shift is likely due to a combination of slight fabrication tolerances during the PCB milling process and potential deviations in the actual dielectric constant of the Rogers RO5870 substrate from the nominal value used in the simulation. The difference in the *S*_11_ magnitude is often associated with the non-ideal characteristics of the manually soldered SMA connector and its transition, which can be challenging to model with perfect accuracy. Despite these small, explainable discrepancies, the excellent overall agreement between the simulation and measurement validates the accuracy of the CST model and the soundness of the presented design approach.

**Fig. 8:**
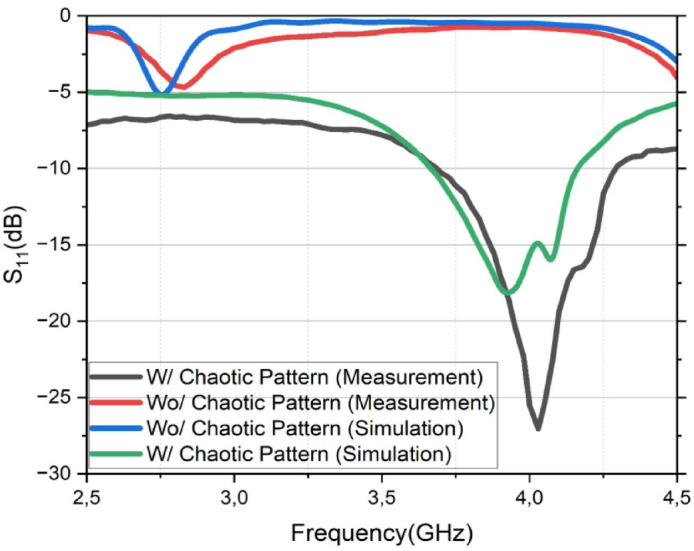
Comparison of simulated and measured reflection coefficient (*S*_11_) results. The strong agreement between the simulated (green) and measured (black) curves for the proposed sensor with the chaotic DGS validates the accuracy of the simulation model.

### Glucose sensing performance with phantoms

The final and most critical phase of the experimental validation involves measuring the sensor’s response to the multi-layer phantoms with varying glucose concentrations. The experimental setup used to acquire the glucose-dependent S-parameter data is shown in Fig. 8. The prepared phantom assembly for each glucose level is carefully placed onto the sensor’s surface, ensuring consistent positioning and contact for each measurement.

**Fig. 9:**
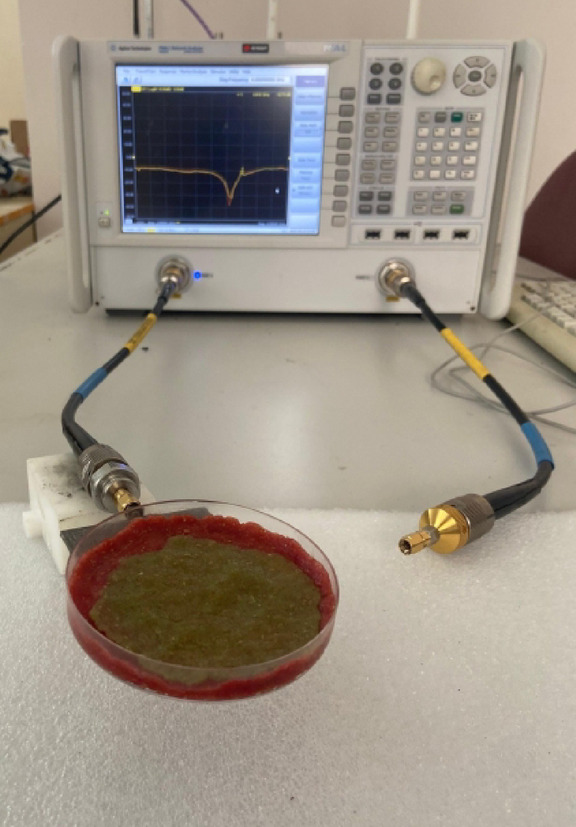
The experimental setup for measuring the glucose sensing performance, showing a prepared multi-layer phan- tom placed on the sensor and connected to the VNA.

The results, presented in Fig. 9, demonstrate the resonance behavior of the sensor in response to different glucose concentrations. The “Air” condition (purple curve), where the sensor is unloaded, serves as a baseline and exhibits a sharp and deep resonance at approximately 4.05 GHz. When the tissue-mimicking phantoms are introduced, the dielectric loading causes the primary resonance to shift significantly upward to a new center frequency of approximately 4.5 GHz.

**Fig. 10:**
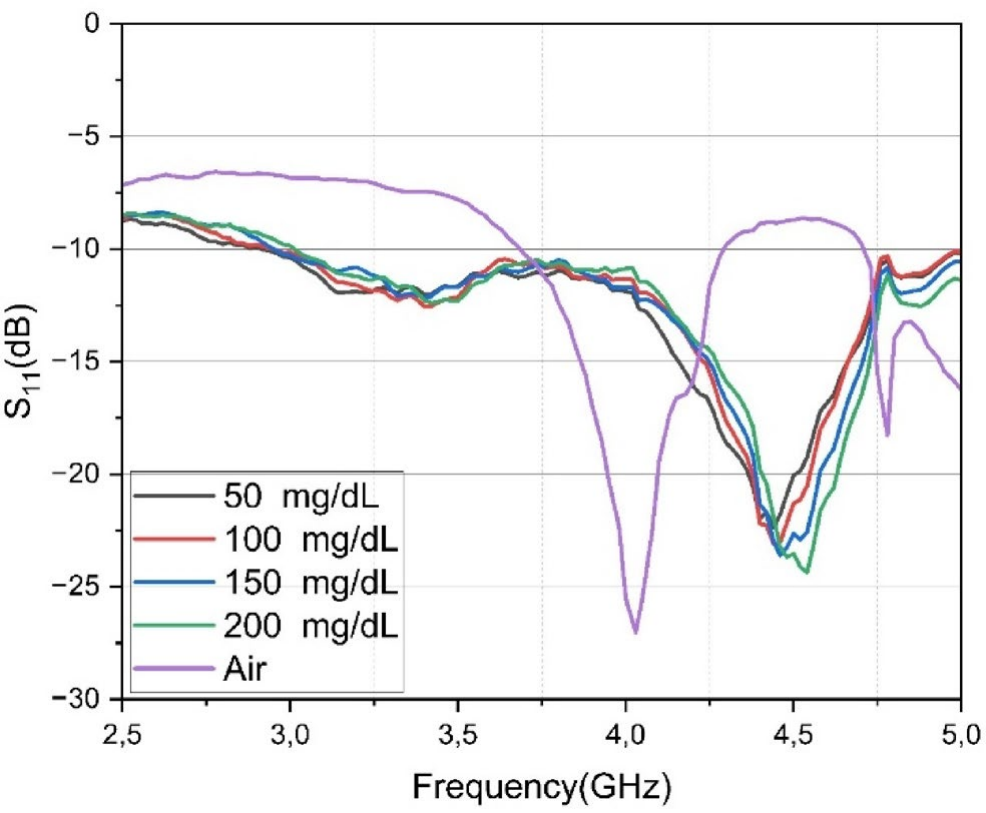
Measured reflection coefficient (S11) of the sensor for multi-layer phantoms with varying glucose concentrations (50, 100, 150, and 200 mg/dL) and for the sensor in air. The results demonstrate a clear shift in resonant frequency and depth in response to changes in glucose levels.

Within this new resonance band, small but consistent frequency shifts are observed for each glucose level. At 50 mg/dL, the resonance frequency appears at its lowest value. With increasing glucose concentration, the resonance is observed to shift upward (to a higher frequency). A more detailed analysis reveals that at the normoglycemic level (100 mg/dL), the resonance shifts to a higher frequency region compared to the hypoglycemic state (50 mg/dL). As glucose concentration further increases to 150 mg/dL and 200 mg/dL (hyperglycemia), the resonance frequency continues to shift upward, and the separation between the curves becomes more pronounced. Particularly at 200 mg/dL, both the position and the depth of the resonance differ significantly, highlighting the strong impact of elevated glucose on the complex permittivity of the blood layer. Thus, the sensor demonstrates a predictable trend of frequency shift in response to incremental increases in glucose, enabling the differentiation of clinically meaningful levels.

Table 2 summarizes the measured resonant frequencies, reflection amplitudes S_11_, and calculated Q-factors for the sensor under air and varying glucose phantom loading conditions. Although the Q-factor decreases compared to air due to the high dielectric loss of the tissue phantom, the increase in glucose concentration from 50 to 200 mg/dL results in a slight improvement in the Q-factor (from 22.99 to 29.20) by reducing the effective loss tangent of the mixture, thus confirming that the sensing resolution remains robust.

**Table 2:** Calculated Q factor values of proposed antenna under different glucose concentrations and air comparasion.

Samples	S_11_ (dB)	F_Resonance_ (GHz)	Q_Factor_
Air	− 27.20	4.04	35.50
50	− 22.369	4.37	22.99
100	− 23.103	4.41	25.63
150	− 23.617	4.44	26.24
200	− 24.368	4.54	29.20

**Fig. 11:**
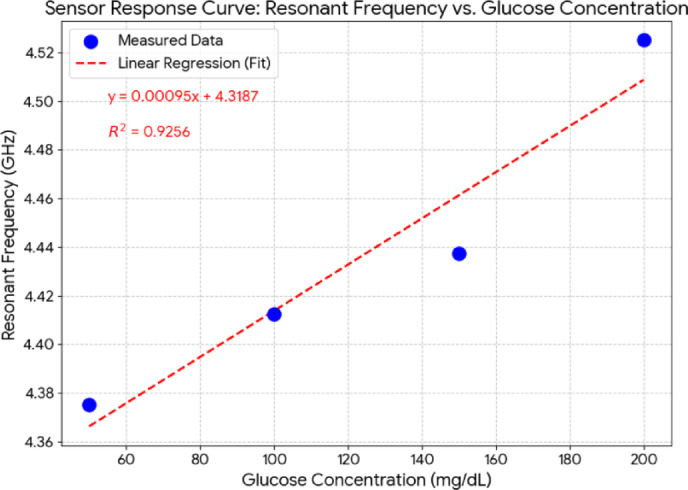
Sensor response curve illustrating the relationship between the resonant frequency and glucose concentration, with data points extracted from the measurements in Fig. 8. A linear regression analysis (red dashed line) is applied to the data, yielding a calculated average sensitivity of 0.950 MHz/(mg/dL) and a coefficient of determination (R²) of 0.9256.

To quantitatively analyze the sensor’s performance, the resonant frequency for each glucose concentration has been determined. As shown in the sensor’s response curve in Fig. 11, a positive correlation has been observed between the glucose concentration and the resonant frequency. A linear regression analysis of the data yields an average sensitivity of 0.950 MHz/(mg/dL) over the 50–200 mg/dL range. The coefficient of determination (R²) has been calculated to be 0.9256, indicating a strong, albeit not perfectly linear, correlation. These quantitative results confirm the sensor’s high performance and its suitability for distinguishing between clinically relevant glucose levels. Furthermore, an examination of the resonance depths shows that strong reflections below − 22 dB have been obtained across all concentrations. This indicates that the sensor can distinguish glucose levels not only through frequency shifts but also by variations in impedance matching.

In conclusion, the obtained measurement results show that the sensor possesses sufficient sensitivity to differentiate between hypoglycemia (50 mg/dL), normoglycemia (100 mg/dL), and hyperglycemia (150–200 mg/dL). This finding strongly validates the reliable performance of the proposed Duffing chaotic DGS-based antenna design within biologically relevant glucose ranges.

## Discussion

The experimental results obtained from the fabricated prototype demonstrate a strong agreement with the simulated model, thereby confirming the accuracy of the design methodology. The integration of the Duffing chaotic attractor-based DGS has shown a decisive role in enhancing both impedance matching and field localization. Compared to the reference antenna with a solid ground plane, the proposed sensor exhibited a sharp resonance at approximately 4.05 GHz with a reflection coefficient of − 27.1 dB, which indicates a highly efficient coupling mechanism.

**Table 3:** Comparison of the proposed sensor with state-of-the-art microwave-based glucose sensors.

Reference	Sensing technology	Operating frequency	testing method	Performance metric(s)	Notes
This Work	Hexagonal Patch with Chaotic Duffing Attractor DGS	~ 4.5 GHz	Multi-layer Aqueous Phantoms	**Sensitivity**: 0.950 MHz/(mg/dL) **Linearity (R²)**: 0.9256	Novel non-Euclidean DGS geometry.
Omer et al.^20^	Honey-cell Hexagonal CSRR	2.4–2.5 GHz	Aqueous Solutions & Preliminary In-vivo (fingertip)	**Sensitivity**: ~0.94 MHz/(mg/dL)	High sensitivity CSRR design. Includes proof-of-concept human test.
Kazemi et al.^24^	Coupled SRR and Patch Resonator	~ 3.3 GHz	In-vitro (microfluidic) &In-vivo (Human Trial)	**Linearity (R²)**: >0.97 (in-vitro) **MARD**: 3.62% (in-vivo)	Advanced study with human trials. Uses S₁₁ amplitude and Machine Learning (LSTM).
Bamatraf et al.^23^	Microwave Dipole Sensor	100–300 MHz	Aqueous Solutions	**Accuracy (RMSE)**: 6.7 mg/dL	Focuses on Machine Learning (GPR) for prediction accuracy. Operates at a much lower frequency.
Tekşen et al.^29^	Transmission Line	2–3 GHz	Aqueous Solutions	**Sensitivity**: dB difference and Frequency shift	DGS geometry and real measurements with transmission line

The superior performance of the chaotic DGS can be attributed to its complex, non-Euclidean geometry. Unlike simple DGS shapes like slots or dumbbells, which introduce localized inductance and capacitance, the fractal-like pattern of the Duffing attractor creates a highly distributed LC network. This forces the surface currents on the ground plane to follow longer, more tortuous paths, leading to a significant increase in equivalent inductance and capacitance. This effect results in a more intense and spatially confined electric field in the near-field region directly above the DGS, where the biological tissue is placed. The enhanced field-tissue interaction makes the sensor’s resonant frequency significantly more sensitive to minute changes in the tissue’s complex permittivity, thus improving the overall glucose detection capability. A simulation of the E-field distribution could further visually confirm this intensified field confinement.

More importantly, the glucose sensing validation using multi-layer tissue-mimicking phantoms confirmed the sensitivity of the sensor across clinically relevant glucose levels. The measured S-parameters revealed a consistent shift in the resonant frequency with increasing glucose concentrations from 50 mg/dL to 200 mg/dL. This behavior directly reflects the dependence of the complex permittivity of blood on glucose content and demonstrates the sensor’s capability to distinguish between hypoglycemic, normoglycemic, and hyperglycemic states.

As detailed in Table 3, the proposed sensor achieves a high sensitivity of 0.950 MHz/(mg/dL), slightly exceeding the ~ 0.94 MHz/(mg/dL) of a state-of-the-art CSRR sensor from Omer et al. This validates the chaotic DGS approach for enhancing intrinsic sensor performance. In contrast to works that focus on machine learning accuracy in lower frequency bands, such as Bamatraf et al. (RMSE of 6.7 mg/dL), our design concentrates on improving physical sensitivity in the GHz band, which is ideal for wearable systems. This phantom-based study establishes a strong foundation for future in-vivo validation, a step demonstrated by Kazemi et al., who achieved a MARD of 3.62% in human trials by analyzing S₁₁ amplitude with advanced machine learning.

While this study successfully demonstrates a proof-of-concept, certain limitations should be acknowledged as avenues for future research. The experiments were conducted on static, tissue-mimicking phantoms under controlled laboratory conditions. In a real-world scenario, factors such as blood flow, body temperature fluctuations, and movement could influence the measurements. Furthermore, this study did not investigate the sensor’s selectivity against other blood components (e.g., lactate, urea, electrolytes) that could also affect the blood’s dielectric properties. The next crucial steps will therefore involve developing more dynamic phantom models, implementing temperature compensation mechanisms, and ultimately, validating the sensor’s performance in clinical trials with human subjects to assess its real-world efficacy and reliability.

It should be noted that minor discrepancies between simulation and measurement, such as frequency shifts on the order of 100 MHz, are primarily attributed to fabrication tolerances, material inhomogeneity in the phantoms, and connector parasitics. Nevertheless, the high level of correlation validates the robustness of the proposed design. Another notable advantage is the choice of the ISM band, which ensured a suitable trade-off between penetration depth and sensitivity, thereby enabling the sensor to achieve both biological relevance and practical applicability for wearable devices.

## Conclusion

In this work, a novel non-invasive glucose sensor based on a hexagonal microstrip patch antenna with a chaotic Duffing attractor-inspired DGS has been presented and experimentally validated. The sensor was carefully optimized to operate in the ISM frequency band, ensuring sufficient electromagnetic penetration while maintaining sensitivity to dielectric variations caused by glucose. Both simulations and measurements demonstrated that the chaotic DGS plays a critical role in achieving sharp resonances and strong field confinement. The fabricated prototype successfully distinguished between four different glucose concentrations (50, 100, 150, and 200 mg/dL) using a realistic multi-layer phantom model mimicking human tissue, achieving a notable average sensitivity of 0.950 MHz/(mg/dL). The results indicate not only high sensitivity but also excellent consistency across the tested glucose range, demonstrating the sensor’s capability to resolve clinically relevant variations. Compared to previously reported non-invasive glucose sensors, the chaotic DGS design exhibits improved resonance stability and stronger tissue penetration, which are essential for practical applications. Moreover, the experimental findings confirm that the chaotic attractor principle enhances the robustness of dielectric sensing by reducing susceptibility to noise and environmental fluctuations. The clear correlation between glucose levels and the measured resonant responses highlights the potential of this approach as a reliable alternative to invasive blood glucose monitoring. These findings suggest that the proposed sensor could significantly improve patient comfort and adherence compared to conventional finger-prick methods. Future work will focus on miniaturizing the design for integration into wearable platforms, improving phantom models with dynamic blood flow simulations, and validating the sensor performance in clinical trials. Overall, the proposed system provides a strong foundation for next-generation, pain-free, and continuous glucose monitoring technologies.

## Data Availability

The data supporting the findings of this study are available from the corresponding author, upon request.
